# Margem de Segurança para Hiperviscosidade Plasmática para Prevenção de Trombose em Pacientes com Doença Cardiovascular após Vacinação contra COVID-19

**DOI:** 10.36660/abc.20210555

**Published:** 2021-11-22

**Authors:** Rujittika Mungmunpuntipantip, Viroj Wiwanitkit

**Affiliations:** 1 Dr DY Patil, Universidade de Pune Pune Índia Dr DY Patil, Universidade de Pune, Pune - Índia

**Keywords:** Keywords, Viscosity, Patients, Margins of Excision

Caro Editor,

Foi com grande interesse que lemos o artigo intitulado “Vacinação do Cardiopata contra COVID-19: As Razões da Prioridade”.^1^ A Fvacinação contra a COVID-19 tem sido considerada a melhor maneira de se prevenir a doença, embora dados sobre sua segurança ainda sejam necessários. Um problema importante após a vacinação contra COVID-19 são as alterações reológicas que induzem a formação de coágulos intravasculares e complicações trombóticas.^2^ Mudanças na viscosidade sanguínea após a vacina da COVID-19 foram confirmadas. A vacina pode induzir uma rápida produção de anticorpo, o qual é uma proteína que pode aumentar a viscosidade do plasma em um valor estimado de 2,4 centipoise (cP) em relação a valores normais em uma pessoa saudável.^3,4^ O problema pode ocorrer se a viscosidade plasmática atingir níveis superiores a 5 cP, o que é considerado um estado de hiperviscosidade.^5^

Para qualquer pessoa que seja vacinada contra a COVID-19, deve existir uma margem de segurança para o problema de hiperviscosidade. Essa margem de segurança seria a diferença entre a viscosidade plasmática basal e o nível de hiperviscosidade. Uma preocupação em relação à margem de segurança para a hiperviscosidade deve ser considerada em relação a indivíduos com doença cardíaca subjacente. Observa-se que uma pessoa com dislipidemia ou doença cardiovascular apresenta uma viscosidade plasmática mais alta em comparação a uma pessoa saudável.^6,7^

Nesse artigo,^1^ os autores tentaram estimar uma margem de segurança para hiperviscosidade plasmática para indivíduos sadios e para aqueles com diferentes doenças cardíacas subjacentes que serão vacinados contra a COVID-19. Os valores estimados estão descritos na Tabela 1; as margens de segurança diferiram-se não somente entre indivíduos sadios e pacientes com doenças cardiovasculares, como entre diferentes doenças cardiovasculares. Uma pessoa com angina instável parece apresentar o maior risco. Pacientes com algumas doenças subjacentes podem apresentar valores mais baixos de margem de segurança, o que pode resultar em um risco maior de se desenvolver doença trombótica. Como recomendação, atenção especial deve ser dada a pacientes com doenças cardiovasculares com risco elevado de hiperviscosidade, e monitoramento de valores de viscosidade plasmática antes da vacinação pode ser útil.

**Tabela 1:** Margem de segurança estimada para hiperviscosidade plasmática após vacinação contra COVID-19 em indivíduos sadios e pacientes com diferentes doenças cardiovasculares

Grupos	Valor de viscosidade basal* (cP)	Margem de segurança estimada** (cP)
Indivíduo sadio	1,40	1,1
Paciente com dislipidemia	1,44	1,06
Paciente com angina estável	1,42	1,08
Paciente com angina instável	1,66	0,84
Paciente com infarto do miocárdio	1,53	0,97

* Valor de viscosidade basal derivado de estudos prévios^3,6,7^

** Margem de segurança pode ser estimada por: “ponto de corte para hiperviscosidade -viscosidade plasmática basal - aumento esperado na viscosidade plasmática após vacinação”.

cP: centipoise.
