# Supplementary material for: Margem de Segurança para Hiperviscosidade Plasmática para Prevenção de Trombose em Pacientes com Doença Cardiovascular após Vacinação contra COVID-19
Source: Arq Bras Cardiol. 2021 Nov 22;117(6):1217–8. [Article in Portuguese] doi: 10.36660/abc.20210555 (PMC8757142; doi:10.36660/abc.20210555)
Supplement: Translation

# Translation: Safety Margin for Plasma Hyperviscosity in Cardiovascular Disease Patients after COVID-19 Vaccination for Thrombosis Prevention

**Authors:** Rujittika Mungmunpuntipantip, Viroj Wiwanitkit

**Affiliations:** 1 Pune India Dr DY Patil, Universidade de Pune, Pune - India

**Keywords:** Viscosity, Patients, Margins of Excision

Dear Editor,

We read the article entitled “Vaccinating Patients with Heart Disease Against COVID-19: The Reasons for Priority” with great interest.^1^ COVID-19 vaccination is accepted as the best way for preventing the disease, but data on its safety are still warranted. An important problem found after COVID-19 vaccination is the rheological change causing intravascular clotting and thrombotic complications.^2^ Changes in blood viscosity after COVID-19 vaccination have been confirmed. A vaccine can induced rapid production of antibody, which is a protein that can increase plasma viscosity by an estimated 2.4 centipoise (cP) from normal values in healthy person.^3,4^ The problem might occur if changes in plasma viscosity reach levels above 5 cP, considered a hyperviscosity state.^5^

For any person receiving the COVID-19 vaccine, there may be a safety margin for the hyperviscosity problem. This safety margin would be difference between baseline plasma viscosity to the hyperviscosity level. The concern on the safety margin for the hyperviscosity problem should be discussed for a person with underlying heart disease. It is observed that a person with hyperlipidemia or cardiovascular disease has higher plasma viscosity than a healthy person.^6,7^

In this paper,^1^ the authors tried to estimate the safety margin for plasma hyperviscosity for healthy people and for those with different underlying heart diseases who will be vaccinated against COVID-19. The estimated safety margin values are shown in Table 1; the safety margins differed not only between healthy individuals and cardiovascular disease patients, but also between different cardiovascular diseases. A person with unstable angina seems to have the highest risk. Patients with some specific underlying diseases might have lower safety margin values, which may imply a higher risk for developing a thrombotic disease. As a recommendation, special attention should be given to cardiovascular patients at high risk of hyperviscosity, and pre-vaccination monitoring of plasma viscosity might be useful.

**Table 1:** Estimated safety margin for plasma hyperviscosity after COVID-19 vaccination in healthy individuals and patients with cardiovascular diseases

Groups	Background viscosity * value (cP)	Estimated safety margin** (cP)
Healthy person	1.40	1.1
Patient with dyslipidemia	1.44	1.06
Patient with stable angina	1.42	1.08
Patient with unstable angina	1.66	0.84
Patient with myocardial infarction	1.53	0.97

* Background viscosity values are derived from previous studies.^3,6,7^

** safety margin for hyperviscosity can be estimated by “hyperviscosity cutoff value – baseline plasma viscosity - expected increase in plasma viscosity after vaccination.

cP: centipoise.
